# Peptides: powerful, pervasive, and full of potential

**DOI:** 10.1093/jxb/eraf379

**Published:** 2025-11-12

**Authors:** Alyssa Kearly

**Affiliations:** Boyce Thompson Institute, Cornell University, Ithaca, NY 14850, USA

**Keywords:** Abiotic stress, bioengineering, biotic stress, development, dipeptides, signaling peptides, sORF-encoded peptides

## Abstract

Small peptides are a diverse and biologically integral group of biomolecules that, due to their organic nature and multitude of functions, are poised to be attractive targets for commercialization and bioengineering in a world that must grapple with changes in climate and societal needs. In this Special Issue, discussions of peptides both novel and well-characterized highlight the multifaceted diversity of these functional biomolecules, emphasize that there is still so much to discover, and stress their potential not only for practical applications, but also for shaping our understanding of fundamental biological principles.

Despite their small size, functional peptides constitute a powerful class of biological molecules, diverse in their biogenesis, structural composition, and activity. Some peptides, including many signaling peptide families supported by decades of research, are derived from precursor proteins that undergo post-translational modification (PTM) and targeted proteolysis. Other, arguably less well-studied, peptides are encoded directly in the genome and expressed from short ORFs (sORFs). Still others, namely dipeptides, can be synthesized by enzymes in biochemical reactions completely unrelated to translation. Regardless of how they come to be, peptides have been shown to play critical roles as signaling molecules, macromolecular structural components, and metabolic regulators, impacting virtually all biological processes (Fig. 1).

**Fig. 1.**
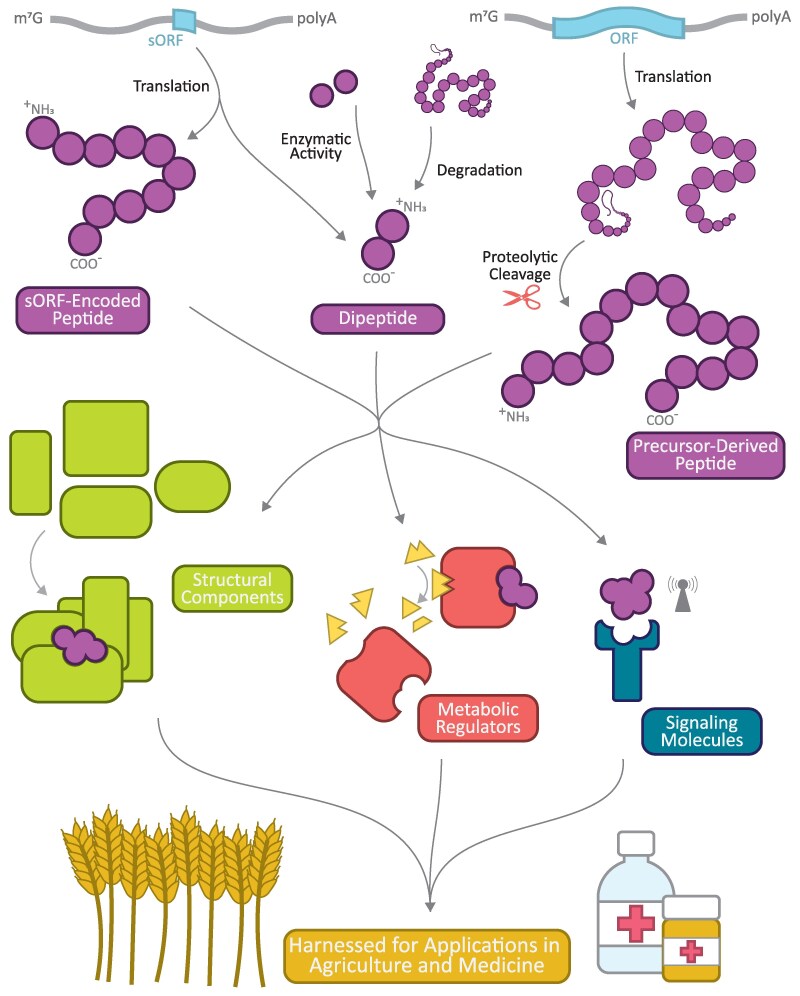
Peptides are a diverse group of biomolecules that can be generated via translation of short open reading frames (sORFs), cleavage of precursor proteins, or, for dipeptides in particular, enzymatically linkage of free amino acids. All of these kinds of peptides can play critical roles as structural components of larger macromolecular complexes, metabolic regulators, and signaling components. With their myriad functions in processes like growth, development, stress responses and beyond, peptides have the potential to be harnessed for agriculture and therapeutics.

## Signaling peptides: well studied and full of potential

The 1991 discovery of systemin, a signaling peptide of 18 amino acids originally found in tomato, revolutionized the field of phytohormones by introducing the idea that plant hormones were not limited to non-protein metabolites, and that small peptides could also deliver local and systemic signals (Pearce *et al.*, 1991). In the decade that followed, several signaling peptide families were identified, and developments in their characterization continue to this day. These peptides are generally encoded in the genome as larger precursors which, after translation, undergo various processing steps to yield the final bioactive peptides. Several well-known signaling peptide families are discussed in this Special Issue through biochemical, molecular, and evolutionary lenses.

Signaling peptides can be categorized into several classes (reviewed in Torres Ascurra and Müller, 2025): PTM peptides, cysteine-rich peptides (CRPs), and non-modified and non-cysteine-rich peptides. PTM peptides are those that require modifications such as tyrosine sulfation [e.g. phytosulfokine (PSK) reviewed by Wu *et al.*, 2025] or proline hydroxylation [e.g. CLAVATA3/EMBRYO SURROUNDING REGION-RELATED peptides (CLE); Cornelis and Hazak, 2025; Perkins *et al.*, 2025; Sen and Bharati, 2025]. CRPs are characterized by their multiple cysteine residues that form disulfide bonds to stabilize secondary structure, and include EPIDERMAL PATTERNING FACTOR (EPF)/EPFL peptides (reviewed by Lu *et al.*, 2025), RAPID ALKALINIZATION FACTOR peptides (RALFs; reviewed by Lu *et al.*, 2025; Perkins *et al.*, 2025), and defensins (investigated by Kalunke *et al.*, 2025). Intuitively, non-modified and non-cysteine-rich peptides meet neither of these criteria (Torres Ascurra and Müller, 2025). Regardless of classification, these peptides all play critical roles in autocrine and intercellular signaling.

The first CLE gene reported in Arabidopsis was CLAVATA3 (CLV3), the mutation of which resulted in a dramatic enlargement of shoot apical meristems due to its role in promoting stem cell differentiation (Clark *et al.*, 1995). In the three decades since, the CLE family has become one of the most well-studied signaling peptide families, with large gene groups identified in species throughout the plant lineage playing diverse roles in development, reproduction, and stress response. Cornelis and Hazak (2025) approach the CLE family from an evolutionary angle in their Expert View, discussing the origins of the peptides in bryophytes and ferns. In describing recent studies on CLE function in these more anciently diverged lineages, the authors highlight the instances in which CLE functions align with and, perhaps more interestingly, diverge from angiosperm CLE function. Within the seed-bearing lineage, CLE peptides have also been investigated in tree species, where their roles in cambial dynamics reflect the functions of Arabidopsis CLEs in meristematic maintenance and vascular differentiation (Sen and Bharati, 2025). In their review, Sen and Bharati (2025) focus on CLE functions in the angiosperms of the *Populus* genus and note the conservation of CLE signaling mechanisms in the more distantly related gymnosperm *Pinus sylvestris*, highlighting a shared regulatory pathway of cambial growth. The authors draw upon evidence from these species as well as Arabidopsis to propose that CLEs could be harnessed to increase wood production and improve wood quality. With ever-continuing efforts to sequence more plant genomes, further insight into the evolution of not only the CLE peptides themselves, but also of CLE signaling pathways and the functional outcomes thereof, is sure to come.

PSK is another PTM peptide with critical biological roles and practical use potential. First isolated in 1996 (Matsubayashi and Sakagami, 1996), the last three decades have seen extensive developments in our understanding of its biogenesis, downstream signaling, and functional characterization, all of which are thoroughly discussed by Wu *et al*. (2025) in this Special Issue. From a biochemical perspective, the authors provide information on the major players involved in the PTM and proteolytic processing of PSKs as they journey from pre-proprotein to bioactive peptide through the secretory pathway, as well as insight into the crosstalk between PSK signaling and that of other phytohormones. Notably, Wu *et al*. (2025) describe the impacts that PSKs can have on agronomically important traits such as crop yield, quality, and stress tolerance, underscoring the idea that these small peptides can be attractive targets for bioengineering and commercialization.

Other peptide families with extensive bodies of supporting research include the RALFs and EPFs/EPFLs of the CRPs, both of which have well-characterized signaling pathways. As reviewed by Lu *et al.* (2025), EPFs/EPFLs are involved in morphogenic processes such as ovule spacing, leaf serration, and stomatal patterning, while RALFs are known to play critical roles in pollen tube formation and responses to biotic and abiotic stress. Synthesizing these aspects of RALF biology, a meta-analysis by Perkins *et al.* (2025) suggests that RALFs may be key players in reproductive thermotolerance. In their larger discussion, the authors provide an in-depth look at the roles of signaling peptides in the various stages of plant reproduction, as well as how heat stress can impact these processes. As increasing global temperatures challenge crop health and yield maintenance, such insights will be crucial to identifying effective solutions.

Defensins are a diverse class of CRPs involved in plant innate immune responses. In this Special Issue, Kalunke *et al.* (2025) build on their previous efforts to characterize defensins in a meticulous examination of the *Medicago truncatula* bi-domain defensin MtDef5. The MtDef5B defensin domain demonstrated fungicidal activity against several different fungal pathogens tested, and, fascinatingly, the authors could optimize the amino acid sequence of an 18 amino acid peptide derived from MtDef5A to increase its fungicidal effectiveness. These peptides have a multi-point mechanism of action, disrupting fungal pathogen plasma membranes and inducing reactive oxygen species to elicit fungal cell death. This confers an advantage over synthetic chemicals predominantly used in biocontrol as they are less likely to induce resistance in the target pathogens.

Defensins are not the only signaling peptides involved in plant–microbe interactions, and extensive efforts have been made in the last decade or so to parse the roles of signaling peptides in achieving balance between permitting the presence of beneficial microbes while maintaining defense against harmful pathogens. In their review, Torres Ascurra and Müller (2025) walk through several major peptide families and detail studies investigating their roles in both pathogenic infection and symbiotic interactions, such as arbuscular mycorrhizal and rhizobial symbioses. Particularly interesting is their discussion on the various ways in which pathogens have developed host plant molecular mimics to facilitate these interactions, adding further complexity to our understanding of symbiosis and pathogen defense. This understanding will be key in developing crops with increased resistance to biotic stress.

## Short open reading frame-encoded peptides: a shadowy but fruitful corner of the proteome

Protein precursors are not the only source of bioactive peptides. Although it is generally accepted that peptides can be directly encoded in sORFs, recent advances in translatomics, the study of the actively translated portion of the transcriptome, have made it clear that functional sORFs encoding bioactive peptides are far more abundant than plant genome annotations would lead us to believe. In particular, sORFs within prototypical non-coding RNAs are translated, challenging our conceptions of what distinguishes a coding gene from a non-coding gene.

In their review, Kearly and Nelson (2025) examine the historical methods of genome annotation and coding versus non-coding classification, as well as the restrictive criteria they employed that have led to this under-representation of sORF-encoded peptide (SEP) genes in the coding population. They make the case that these biases remain pervasive even in the current era of machine learning-fueled coding potential prediction tools, but also highlight recent advances in next-generation sequencing and MS that are helping to shed light on the heretofore dark sORF-encoded peptidome. In addition to drawing attention to long-overlooked SEP genes, the authors emphasize the need for efforts to validate and functionally characterize the large numbers of SEPs commonly identified with these updated techniques, and emphasize the cruciality of continual, reiterative annotation curation.

Directly underscoring the themes discussed by Kearly and Nelson (2025), an Expert View by Thuleau *et al.* (2025) highlights a class of SEPs derived from primary miRNA transcripts that were historically considered non-coding. These so-called miPEPs were originally identified in Arabidopsis and have since been detected in various species across the plant kingdom, as well as in humans, mice, and *Drosophila*. In their review, the authors focus on plant miPEPs and their common effect on the expression of the primary miRNA transcript itself, the processed functional miRNA, and the miRNA target genes. Although it is not discussed in this focused review, it is interesting to note that the miPEPs identified in mammalian species play myriad biological roles beyond promotion of primary miRNA transcript expression (Dozier and Plaza, 2022), potentially suggesting a diversification of miPEP function throughout evolution. Overall, miPEPs constitute a perfect demonstration of the wealth of knowledge and uncharacterized bioactive SEPs hidden in the non-coding portion of the genome.

## Dipeptides: compositionally simple, functionally complex

Two amino acids joined by a peptide bond constitute the smallest possible peptide but, despite their small size, these dipeptides play critical biological roles. Seemingly simple, this class of molecules is structurally diverse, comprised of both proteinogenic and non-proteinogenic amino acids with the potential for cyclization. They can be derived from sORFs like SEPs, from the proteolysis of larger proteins such as signaling peptides, or uniquely, they can have a dedicated biosynthetic pathway. In their review, Agarwal *et al.* (2025) dive deep into the intricacies of dipeptide biogenesis, composition, and function. From metabolism to stress responses, dipeptides interact with larger proteins and impact an array of biological processes. As the authors note, cyclic dipeptides of microbial origin can influence pathogen–host plant interactions, providing an interesting expansion on the discussion by Torres Ascurra and Müller (2025) of immune-related signaling peptides.

## Power in peptides: applications for a changing society

A common theme emphasized throughout this Special Issue is the potential for peptides to be harnessed in agriculture. With an ever-changing climate and decreasing food security, there is a pressing need for the development of more resilient, higher yielding crops that can better meet the needs of society. As key regulators of growth, reproduction, and responses to biotic and abiotic stresses, peptides constitute promising targets for bioengineering, and their organic nature makes them prime candidates for use in the field as fertilizers, weed managers, and resilience and immunity supplements.

Plants in the field can be exposed to a plethora of pathogens that challenge their carefully curated homeostasis, negatively impacting crop yield and threatening food security. To combat these challenges, researchers are looking to harness antimicrobial and immunomodulatory peptides. Defensins have broad-spectrum antimicrobial activity, varying mechanisms of action, and structural stability in a wide range of chemical conditions, making them attractive targets for these efforts. Indeed, in their investigations reported here, Kalunke *et al.* (2025) found that application of MtDef5-derived peptides was effective in preventing and controlling gray mold infections and stymying anthracnose fruit rot. Additionally, PSK or RALF dysregulation and exogenous application of certain miPEPs can modulate the host plant immune response during different pathogen infections (Lu *et al.*, 2025; Thuleau *et al.*, 2025; Torres Ascurra and Müller, 2025; Wu *et al.*, 2025). These findings highlight the power of harnessing natural plant products to engineer effective biocontrol agents, either by eliminating the pathogenic threat or by boosting the host immune response.

Peptides can also promote crop growth and survival. Thuleau *et al*. (2025) note several examples of miPEP application leading to increased root growth and abiotic stress tolerance. CLE peptides are well known for their roles in growth and stress responses (Perkins *et al*., 2025), though functional redundancy and low physical stability could impede their practical application in bioengineering and agrochemicals (Sen and Bharati, 2025). Moreover, PSK has a host of potential agricultural implications, having been shown to impact growth, yield, nutritional content, and stress responses (Wu *et al*., 2025). Of particular interest, PSK has also been shown to increase transformation efficiency (Wu *et al*., 2025), which could accelerate the translation of research from the lab to farmers’ fields. Work is continually being done to identify bioengineering targets that can improve crops, but a major barrier to its practical application is the difficulty in genetically altering commercial crop species. It seems that peptides could be tools as well as targets for bioengineering.

## Conclusions

Peptides are an incredibly diverse class of biomolecules, and we have probably only just begun to uncover the full extent of their existence and function. Well-established signaling peptide families have been studied for decades, though there is still much to learn about their intricate signaling pathways and context-specific impacts. Beyond sequence conservation, it will be interesting to learn more regarding the functional evolution of these peptides as more genomic resources become available for more species. Also compelling is the potential wealth of unexplored SEPs hidden in the supposedly non-coding portion of the genome. When considering the molecular functions and biological roles to be discovered in this population, the possibilities are boundless. This Special Issue serves to celebrate the small but mighty peptides that, though often easy to overlook, are key components of the plant proteome. Whether they represent large, conserved signaling peptide families or recently uncovered functional SEPs, peptides are powerful biomolecules with massive potential.

## References

[eraf379-B1] Agarwal P, Fischer HD, Camalle MD, Skirycz A. 2025. Not to be overlooked: dipeptides and their role in plant stress resilience. Journal of Experimental Botany 76, 5738–5747. 40628532 10.1093/jxb/eraf311 PMC12516527

[eraf379-B2] Clark SE, Running MP, Meyerowitz EM. 1995. *CLAVATA3* is a specific regulator of shoot and floral meristem development affecting the same processes as *CLAVATA1*. Development 121, 2057–2067.

[eraf379-B3] Cornelis S, Hazak O. 2025. CLE pathways in plant development: recent advances and future perspectives. Journal of Experimental Botany 76, 5748–5754. 40660820 10.1093/jxb/eraf321 PMC12605765

[eraf379-B4] Dozier C, Plaza S. 2022. Functions of animal microRNA-encoded peptides: the race is on! EMBO Reports 23, e54789. 35343609 10.15252/embr.202254789 PMC9066062

[eraf379-B5] Kalunke RM, Pokhrel A, Tetorya M, Godwin J, Nath VS, Czymmek KJ, Shah DM. 2025. Modes of action and bio-fungicide potential of peptides derived from the *Medicago truncatula* bi-domain defensin MtDef5. Journal of Experimental Botany 76, 5755–5769. 40259545 10.1093/jxb/eraf166

[eraf379-B6] Kearly A, Nelson ADL. 2025. Hiding in plain sight: advances in discovery and functional description of plant sORF-encoded peptides. Journal of Experimental Botany 76, 5698–5712. 40576250 10.1093/jxb/eraf240

[eraf379-B7] Lu R, Lanooij J, Smakowska-Luzan E. 2025. From roots to reproduction: the multifaceted roles of rapid alkalinization factor and epidermal patterning factor peptides in plants. Journal of Experimental Botany 76, 5713–5727. 40624948 10.1093/jxb/eraf303 PMC12605736

[eraf379-B8] Matsubayashi Y, Sakagami Y. 1996. Phytosulfokine, sulfated peptides that induce proliferation of single mesophyll cells of *Asparagus officinalis* L. Proceedings of the National Academy of Sciences, USA 93, 7623–7627. 10.1073/pnas.93.15.7623 PMC38796 8755525

[eraf379-B9] Pearce G, Strydom D, Johnson S, Ryan CA. 1991. A polypeptide from tomato leaves induces wound-inducible proteinase inhibitor proteins. Science 253, 895–897. 17751827 10.1126/science.253.5022.895

[eraf379-B10] Perkins CJ, Pryze K, Palanivelu R. 2025. Peptide signaling in flowering plants: insights into reproductive thermotolerance. Journal of Experimental Botany 76, 5666–5681. 40350712 10.1093/jxb/eraf192

[eraf379-B11] Sen MK, Bharati R. 2025. Climate-smart trees: CLE small signaling peptides shaping future forests. Journal of Experimental Botany 76, 5634–5639. 40292602 10.1093/jxb/eraf093

[eraf379-B12] Thuleau P, Ormancey M, Plaza S, Combier J-P. 2025. miPEPs and cPEPs as tools to monitor plant gene expression and develop alternative strategies in agriculture. Journal of Experimental Botany 76, 5728–5737. 39688938 10.1093/jxb/erae501

[eraf379-B13] Torres Ascurra YC, Müller LM. 2025. Signaling peptides control beneficial and pathogenic plant–microbe interactions. Journal of Experimental Botany 76, 5640–5665. 40320570 10.1093/jxb/eraf180

[eraf379-B14] Wu C, Munir R, Li F, Li P, Cai Y, Shi K. 2025. From biosynthesis to signaling: unveiling the multifaceted roles of phytosulfokine peptide in plants. Journal of Experimental Botany 76, 5682–5697. 40405478 10.1093/jxb/eraf230

